# No effect of short-term amino acid supplementation on variables related to skeletal muscle damage in 100 km ultra-runners - a randomized controlled trial

**DOI:** 10.1186/1550-2783-8-6

**Published:** 2011-04-07

**Authors:** Beat Knechtle, Patrizia Knechtle, Claudia Mrazek, Oliver Senn, Thomas Rosemann, Reinhard Imoberdorf, Peter Ballmer

**Affiliations:** 1 Gesundheitszentrum St. Gallen, St. Gallen, Switzerland; 2 Institute of General Practice and Health Services Research, University of Zurich, Zurich, Switzerland; 3 Departement Medizin, Klinik für Innere Medizin, Kantonsspital Winterthur, Switzerland

## Abstract

**Background:**

The purpose of this study was to investigate the effect of short-term supplementation of amino acids before and during a 100 km ultra-marathon on variables of skeletal muscle damage and muscle soreness. We hypothesized that the supplementation of amino acids before and during an ultra-marathon would lead to a reduction in the variables of skeletal muscle damage, a decrease in muscle soreness and an improved performance.

**Methods:**

Twenty-eight experienced male ultra-runners were divided into two groups, one with amino acid supplementation and the other as a control group. The amino acid group was supplemented a total of 52.5 g of an amino acid concentrate before and during the 100 km ultra-marathon. Pre- and post-race, creatine kinase, urea and myoglobin were determined. At the same time, the athletes were asked for subjective feelings of muscle soreness.

**Results:**

Race time was not different between the groups when controlled for personal best time in a 100 km ultra-marathon. The increases in creatine kinase, urea and myoglobin were not different in both groups. Subjective feelings of skeletal muscle soreness were not different between the groups.

**Conclusions:**

We concluded that short-term supplementation of amino acids before and during a 100 km ultra-marathon had no effect on variables of skeletal muscle damage and muscle soreness.

## Background

Apart from the classical marathon distance of 42.195 km, an increasing number of studies of athletes participating in ultra-marathons over 100 km [1-3] or further [4-6] has been published in recent years. Based on the high eccentric demands of these activities, marathon and ultra-marathon running as eccentric exercise lead to skeletal muscle damage resulting in an increase in myocellular enzymes such as plasma creatine kinase [1,4,6], urea [3,7,8], and myoglobin [1,7,9].

It has been shown that the breakdown of body protein during endurance exercise occurs and the mobilized amino acids are available for increased rates of oxidation and gluconeogenesis during endurance performances [10]. The increase in variables of skeletal muscle damage during ultra-endurance running might be associated with the decrease in skeletal muscle mass as has been shown in ultra-marathoners [2,11,12].

In recent years, several laboratory studies in cyclists reported reductions of myocellular enzymes indicative of skeletal muscle damage during endurance performances, and enhanced performance after combined ingestion of carbohydrates and protein. It has been demonstrated that consumption of a carbohydrate-protein beverage during an intense cycling performance led to a reduced increase in plasma creatine kinase [13,14] and myoglobin [15]. Subjects were given 200 ml of a carbohydrate (6%) or carbohydrate plus casein hydrolysate (6% carbohydrate + 1.8% protein hydrolysate) 500 ml immediately pre-exercise and every 5 km in the study of Saunders *et al. *[15]. In the study of Valentine *et al. *[15], participants consumed 250 ml placebo, carbohydrates (7.75%), carbohydrate plus carbohydrates (9.69%) or carbohydrates plus protein (7.76% + 1.94%) every 15 min until fatigue. The combined intake of carbohydrate and protein enhanced cycling performance [16,17] and reduced ratings of muscle soreness [14]. The ingestion of amino acids before a performance reduced both delayed onset of muscle soreness and muscle fatigue for several days after exercise [18]. In addition, it was discovered that amino acid supplementation during training prevented exercise induced muscle proteolysis [19].

To date, no study has investigated whether the supplementation of amino acids would have an effect on variables of skeletal muscle damage and performance in ultra-endurance runners competing in events further than the classic marathon distance. We therefore asked whether the short-term supplementation of amino acids before and during a 100 km ultra-marathon might have an effect on variables of skeletal muscle damage in ultra-endurance athletes. Regarding the present literature, we hypothesized that the supplementation of amino acids before and during an ultra-marathon would lead to a reduced increase in the variables of skeletal muscle damage, a decrease in muscle soreness and an improved performance.

## Methods

An interventional field study at the '100 km Lauf Biel' in Biel, Switzerland was used for this research. The organizer contacted all participants of the race in 2009 via a separate newsletter at the time of inscription to the race, in which they were asked to participate in the study. About 1,000 male Caucasian runners started in the race; a total of 30 male ultra-runners volunteered to participate in this investigation. This study was approved by the Institutional Review Board for use of Human Subjects of the University of Berne, Switzerland.

### Subjects

A total of 28 athletes participated in this investigation. Table 1 represents the anthropometric data for the participants, Table 2 their pre-race training variables. The athletes were informed of the experimental risks and gave their informed written consent.

**Table 1:** Comparison of pre-race age and anthropometry of the participants

	Amino acids (n = 14)	Control (n = 14)
Age (years)	42.4 (9.1)	45.1 (6.1)
Body mass (kg)	72.1 (6.4)	75.1 (5.6)
Body height (m)	1.74 (0.06)	1.80 (0.06)
Body mass index (kg/m^2^)	23.5 (1.5)	22.9 (2.2)
Percent body fat (%)	14.1 (3.0)	16.0 (4.5)

Results are presented as mean (SD). No significant differences were found between the two groups.

**Table 2:** Comparison of pre-race training and experience of the participants

	Amino acids (n = 14)	Control (n = 14)
Years as active runner	13.1 (9.4)	10.3 (8.3)
Average weekly running volume (km)	81.6 (21.8)	60.0 (16.2)
Average weekly running volume (h)	7.4 (2.3)	5.7 (2.0)
Average speed in running during training (km/h)	10.9 (1.8)	11.2 (1.1)
Number of finished 100 km runs	5.7 (5.1) (n = 10)	2.8 (2.3) (n = 8)
Personal best time in a 100 km run (min)	601 (107)	672 (98)

Results are presented as mean (SD). No significant differences were found between the two groups.

### Measurements and Calculations

Ultra-runners volunteering for this investigation kept a comprehensive training dairy, including recording their weekly training units in running, showing duration (minutes) and distance (kilometres), from inscription to the study until the start of the race. In addition, they reported their number of finished 100 km runs including their personal best time in a 100 km. ultra-marathon. The personal best time was defined as the best time the athletes ever had achieved in their active career as an ultra-runner.

The athletes who agreed to participate were randomly assigned to the amino acid supplementation group or the control group upon inscription to the study. In case an athlete withdrew, the next athlete filled the gap. Twenty-eight of the expected 30 athletes reported to the investigators at the race site, between 04:00 p.m. and 09:00 p.m. on June 12 2009.

The athletes in the group using amino acid supplementation received, on the occasion of the pre-race measurements, a pre-packed package of amino acids in the form of a commercial brand of tablets (amino-loges^®^, Dr. Loges + Co. GmbH, 21423 Winsen (Luhe), Germany). The composition of the product is represented in Table 3. These athletes ingested 12 tablets one hour before the start of the race, and then four tablets at each of the 17 aid stations. The runners took a total of 80 tablets in the pockets of their race clothing. In total, they ingested 52.5 g of amino acids; 20 g were branched-chain amino acids. During the run, they consumed food and fluids at the aid stations *ad libitum*. At each aid station, they recorded their intake of nutrition and fluid. Due to the manufacturer's concerns regarding the high calcium content of the placebo tablets which, in combination with an expected dehydration, could be harmful for the renal function of the athletes, we had to resign from a placebo control. Thus the athletes randomly assigned to the control group also consumed food and fluids *at libitum *and recorded their nutrient and fluid intake, but did not receive any placebo tablets.

**Table 3:** Composition of the amino acid supplementation

Amino acid	Per Tablet (mg)	During the whole race (g)
L-Leucine	125	10
L-Ornithine	62.5	5
L-Isoleucine	62.5	5
L-Valine	62.5	5
L-Arginine	62.5	5
L-Choline	31.25	2.5
L-Cysteine	50	4
L-Tyrosine	50	4
L-Lysine	31.25	2.5
L-Phenylalanine	31.25	2.5
L-Threonine	31.25	2.5
L-Histidine	31.25	2.5
L-Methionine	12.5	1
L-Tryptophan	12.5	1

Twenty-eight of the expected 30 athletes reported, between 04:00 p.m. and 09:00 p.m. on June 12 2009 to the investigators for their pre-race anthropometric measurements and the collection of blood samples. Upon arrival at the finish, the same measurements were performed within one hour after finishing, there being 27 finishers.

#### Questionnaires of subjective feelings

In combination with the pre- and post-race measurements, the athletes were asked about their subjective feelings of muscle soreness, using a subjective 0-20 scale from 0 (absolutely no muscle soreness) to 20 (highest subjective discomfort with muscle soreness). After the race, the athletes were asked whether they had performed the run as expected, weaker than expected or better than expected.

#### Anthropometric measurements

Body mass was measured using a commercial scale (Beurer BF 15, Beurer GmbH, Ulm, Germany) to the nearest 0.1 kg. Body height was determined using a stadiometer to the nearest 1 cm. Body mass index (kg/m^2^) was calculated using body mass and body height.

The percentage of body fat was estimated using the following anthropometric formula according to Ball *et al*.: Percent body fat = 0.465 + 0.180 * (Σ7SF) - 0.0002406 * (Σ7SF)^2 ^+ 0.0661 * (age), where Σ7SF = sum of skin-fold thickness of pectoralis, axilla, triceps, sub scapular, abdomen, suprailiac and thigh [20]. Skin-fold data were obtained using a skin-fold caliper (GPM-Hautfaltenmessgerät, Siber & Hegner, Zurich, Switzerland) and recorded to the nearest 0.2 mm. One trained investigator took all the anthropometric measurements in order to eliminate inter-tester variability. The skin-fold measurements were taken once for the entire eight skin-folds and were then repeated twice more by the same investigator; the mean of the three times was then used for the analyses. The timing of the taking of the skin-fold measurements was standardized to ensure reliability, and the readings were performed after 4 s following Becque *et al. *[21].

#### Analysis of blood samples

After venipuncture of an antecubital vein in the right arm while the participants were seated, two Sarstedt S-Monovettes (serum gel, 7.5 ml) for chemical analysis were drawn. Monovettes for serum were centrifuged at 3,000 g for 10 min at 4°Celsius. The serum was collected, stored on ice and transported immediately after collection to the laboratory for analysis within 6 hours. In the serum, urea, creatine kinase, and myoglobin were measured using COBAS INTEGRA^® ^800 (Roche, Mannheim, Germany).

#### Estimation of energy intake and energy expenditure

During the run, the athletes consumed food and drinks *ad libitum *and reported their intake of fluids and solid nutrition at each aid station. At these aid stations, liquids and food such as hypotonic sports drinks, tea, soup caffeinated drinks, water, bananas, oranges, energy bars and bread were prepared in a standardized manner, i.e. beverages and food were provided in standardized size portions. The drinking cups were filled to 0.2 L; the energy bars and the fruits were halved. Ingestion of fluids and solid food were determined according to the reports of the athletes using a food table [22]. Energy expenditure during the event was estimated using body mass, mean velocity and time spent running [23].

### Statistical Analyses

The Shapiro-Wilk test was used to check for normality distribution. Data is presented as mean and standard deviation (mean ± SD). Parametric- and non-parametric, both within a group (pre-compared to post-race) and between groups (differences during the race between the supplementation and control group), comparisons were performed as appropriate. Correlation analyses were applied in order to investigate the effect of the amino acid supplementation on the variables of skeletal muscle damage and changes in anthropometry. In addition we calculated Cohen's ƒ^2 ^as an appropriate effect size that can be applied in the context of multiple regressions to estimate the relative importance of the differences between the two groups. By convention, ƒ^2 ^effect sizes of 0.02, 0.15, and 0.35 are termed small, medium, and large, respectively [24]. Fisher's exact test was applied for categorical data to assess the effect of amino-acid supplementation on the subjective estimation of race outcome. Statistical significance was set at a two-sided p-level < 0.05 for all comparisons.

## Results

Baseline characteristics with regard to anthropometry (Table 1) training and pre-race experience (Table 2) showed no differences between the athletes receiving amino acid supplementation and the control group.

### Performance

One athlete in the control group dropped out after 71 km due to medical problems. Mean (±SD) finishing time of the 14 athletes in the amino acid group was 624.3 (79.5) min., whereas the remaining 13 athletes out of the control group finished in 697.8 (89.7) min. The mean difference of 73.6 min. in race time between the two groups was statistically significant (*p *= 0.033). The corresponding 95% confidence limits of the race time difference were between 6.5 min. and 140.6 min. Race time was significantly associated with personal best time in a 100 km ultra-marathon for both the supplementation and the control group, with Pearson correlation coefficients of 0.77 and 0.81 (*p *< 0.05 for both), respectively. The corresponding mean (95% CI) difference in personal best time between the groups was 71.0 (-33.2 to 175.1) min (*p *= 0.17). Due to the similar mean differences in race time and personal best time in a 100 km ultra-marathon between the two groups, and the significant association between the race time and the personal best time in a 100 km ultra-marathon, we performed a linear regression controlling for personal best time in a 100 km ultra-marathon as a potential confounder for the difference between 100 km race times. The resulting mean (SE) race time difference of 5.5 (±28.6) min. remained no longer statistically significant when adjusted for the personal best time in a 100 km ultra-marathon.

### Energy balance and fluid intake

The athletes in the amino acid group consumed 8.5 (±3.2) L of fluids during the run, the runners in the control group 7.9 (±3.5) L (*p *> 0.05). Energy intake, energy expenditure and energy balance were not different between the two groups (Table 4). The athletes in the amino acid group ingested significantly more protein compared to the control group. The energy deficit was significantly related to the decrease in body mass of the runners in the amino acid group (Pearson *r *= 0.7, *p *= 0.003). The additional effect (Cohen's ƒ^2^) of the amino acid supplementation on the association between the loss of body mass and the energy deficit was 0.018. In the amino acid group, body mass decreased by 1.8 (±1.6) kg, in the control group by 1.9 (±2.0) kg (*p *> 0.05). No associations between the 100 km race time and the change in body mass have been observed in the two groups.

**Table 4:** Comparison of energy balance and nutrient intake of the participants during the race

	Amino acids (n = 14)	Control (n = 13)
Energy expenditure (kcal)	7,160 (844)	7,485 (621)
Energy intake (kcal)	3,311 (1,450)	2,590 (1,334)
Energy balance (kcal)	- 3,848 (1,369)	- 4,894 (1,641)
Intake of carbohydrates (g)	755.7 (354.8)	608.8 (326.4)
Intake of protein (g)	79.9 (12.7) **	26.7 (22.0)
Intake of fat (g)	5.1 (4.8)	7.0 (7.1)

Results are presented as mean (SD). Athletes in the amino acid group ingested highly significantly more protein compared to the control group. ** = *p *< 0.01.

### Changes in serum variables

Plasma concentrations of creatine kinase, urea and myoglobin decreased significantly in the two groups (Table 5). The changes from post- to pre-race (Δ) were no different between the two groups. The post-race values for creatine kinase, serum urea and myoglobin were 2,637 (±1,278) %, 175 (±32) %, and 14,548 (±8,522) % higher than the pre-race values in the amino acid group; and 2,749 (±1,962) %, 168 (±38) %, and 13,435 (±10,724) % in the control group (*p *< 0.01). The increases were not different between the two groups.

**Table 5:** Comparison of changes of blood variables during the race within and between the two groups

	Amino acids (n = 14)			Control (n = 13)			Difference between changes
	**Pre race**	**Post race**	**Δ (post - pre race)**	**Pre race**	**Post race**	**Δ (post - pre race)**	**(Δ amino acids - Δ control)**

**Creatine kinase (U/l)**	168.3 (61.7)	4,582.5 (3,150.3)	4,414 (3,107) **	157.8 (74.5)	3,861.5 (2,357.8)	3,703 (2,340) **	711 (1,065)
**Urea (mmol/l)**	6.2 (1.4)	10.6 (2.1)	4.4 (1.6) **	5.9 (1.5)	9.5 (1.6)	3.6 (1.5)**	0.8 (0.6)
**Myoglobin (μg/l)**	50.2 (17.8)	6,933 (4,208)	6,883 (4,206) **	43.8 (13.0)	5,709 (4,053)	5,665 (4,049) **	1,218 (1,591)

Results are presented as means (SD) for within group comparisons and as means (SE) for between group comparisons; * = *p *< 0.05; ** = *p *< 0.001, respectively for within group comparisons. No significant differences were found when the Δ between the two groups was compared.

In the amino acid group, race time was positively correlated to the increase in plasma urea concentration (Pearson *r *= 0.56, *p *= 0.038), which was not the case in the control group (Pearson *r *= -0.30, *p *= 0.3). The corresponding effect size (Cohen's ƒ^2^) for the observed difference between the race time and the change in urea concentration between the two groups was 0.23.

### Subjective feelings of muscle soreness and performance

In the amino acid group, the subjective feeling of muscle soreness increased from 0.9 (±2.2) pre-race to 11.3 (±4.3) post-race (*p *< 0.05); in the control group from 0.4 (±1.0) pre-race to 9.4 (±4.6) post-race (*p *< 0.05). The changes between the two groups were not different. When the athletes were asked, post-race, whether they had completed the race as expected, better than expected or worse than planned, no differences were found.

## Discussion

In the present study, we have investigated the potential effects of a short term amino-acid supplementation on variables of skeletal muscle damage in ultra-runners during a 100 km ultra-marathon. We hypothesized that the supplementation of amino acids before and during an ultra-marathon would reduce the increase in the variables of skeletal muscle damage, decrease the subjective feeling of muscle soreness and improve race performance. In contrast to our hypothesis, the amino acid supplementation showed no effect on variables of skeletal muscle damage, i.e. creatine kinase and myoglobin, on subjective feelings of muscle soreness and on performance. Potential explanations for these negative findings could be the time and duration of amino acid supplementation and the type of exercise.

### Change in variables of skeletal muscle damage

We hypothesized that an amino acid supplementation would lower post-race values of variables of skeletal muscle damage compared to control participants. In contrast, we found no differences in the increase in serum concentrations of creatine kinase, urea and myoglobin between the two groups. Cockburn *et al. *demonstrated that creatine kinase and myoglobin increased to a lower extent after supplementation with a milk-based protein [25]. However, they measured creatine kinase and myoglobin 24 h and 48 h after exercise, which might explain the disparate findings.

In marathon runners, post-race creatine kinase was significantly elevated among faster compared to slower runners and the elevations of creatine kinase drawn 24 hours after a marathon were inversely related to the finishing times [26]. Skenderi *et al. *described 39 runners in the Spartathlon, a 246 km ultra-marathon, which the athletes completed within 33.3 (±0.5) h [6]. The finishing time was not correlated to the post race creatine kinase concentration, as has been found in the present study.

### Duration of amino acid supplementation

Our athletes ingested the amino acids as a pre-race load of 12 g and then 4 g at each aid station during the 100 km ultra-marathon. The total amount was 52.5 g amino acids and the time of supplementation was between 12 and 13 hours. This time period might be too short to show an effect of the amino acid supplementation on performance. An amino acid supplementation period of two weeks [27], four weeks [28] or even eight weeks [29] showed beneficial effects on performance. The supplementation of amino acids for a shorter period may nonetheless have positive effects on serum variables or muscle soreness. Shimomura *et al. *demonstrated that the ingestion of 5 g of branched-chain amino acids 15 minutes prior to seven sets of 20 squats per set reduced the delayed onset of muscle soreness and muscle fatigue for several days after exercise [18].

The duration of supplementation might also have been too short to show an effect on creatine kinase. Consuming 12 g of branched-chain amino acids during seven days reduced the increase of creatine kinase and lactate dehydrogenase after performance [30]. Ohtani *et al. *showed a decrease in post exercise creatine kinase serum concentrations compared to pre-exercise when athletes ingested, three times per day, 2.2 g of a mixture of amino acids during one month [28]. However, there is data that shows that the ingestion of amino acids during performance has an effect on variables of skeletal muscle damage. In a recent study in untrained male cyclist, the ingestion of branched-chain amino acids reduced the increase in creatine kinase serum concentration after performance [31]. The disparate findings might be explained by the fact that those researchers investigated untrained subjects during cycling where as we investigated well-trained and experienced ultra-runners.

Two recent studies showed an enhanced performance when both protein and carbohydrates were ingested during endurance performances. In two studies of cyclists, the combined intake of carbohydrate and protein during exercise enhanced performance [16,17]. In the first study of Saunders *et al.*, the subjects were given a carbohydrate and protein beverage with 7.3% carbohydrate and protein plus 1.8% protein concentrate versus a carbohydrate-only beverage with 7.3% carbohydrate [16]. In the second study of Saunders *et al.*, the subjects received at 15 min intervals carbohydrate or carbohydrate and protein gels which were matched for carbohydrate content with 0.15 g carbohydrates·kg body mass^-1 ^for the carbohydrate group versus 0.15 g carbohydrates + 0.038 g protein·kg body mass^-1 ^for the carbohydrate plus protein group [17]. In contrast to these findings, four studies demonstrated no improved performance after protein supplementation. In three studies using cyclists [13,32,33] and one study using runners [34], the intake of carbohydrate and protein did not enhance performance compared to carbohydrate intake. In accordance with our findings we must assume that protein supplementation during endurance exercise has no effect on performance.

### Amino acid supplementation and muscle soreness

We hypothesized that the subjective feelings of muscle soreness after the race would decrease while ingesting amino acids. In cyclists, the combined intake of carbohydrate and protein during performance led to significant reductions in muscle soreness compared to carbohydrate intake alone [14]. The supplementation with amino acids before and after elbow flexion lowered muscle soreness in the recovery phase [35]. In a study with branched-chain amino acid supplementation during performance, the subjects' ratings of perceived exertion were 7% lower when branched-chain amino acids were given compared to controls [36]. In contrast to these findings, amino acid supplementation showed no effect on muscle soreness in our ultra-runners. This might be explained by the fact that we have investigated runners and not cyclists [14] and asked for subjective feelings of muscle soreness immediately upon arrival at the finish line, compared to the recovery phase [35].

### Limitations of the present study and implications for future research

The finding that athletes in the amino acid group were significantly faster compared to the control group was not brought about by the ingestion of amino acids but by the study sample. Although the athletes were randomly assigned to the two groups and no statistically significant differences regarding anthropometry and pre-race experience were found between the two groups, we a ssume a potential confounding caused by the personal best time in a 100 km ultra-marathon. The mean difference of 73.6 min. in race time between the two groups was statistically significant. The corresponding 95% confidence limits of the race time difference were between 6.5 min. and 140.6 min. The race time was significantly associated with the personal best time in a 100 km ultra-marathon for both groups. The corresponding mean (95% CI) difference in personal best time between the two groups was 71.0 (-33.2 to 175.1) min (*p *= 0.17). Due to the similar mean differences in race time and personal best time over 100 km between the two groups, and the significant association between the race time and the personal best time in a 100 km ultra-marathon, linear regression controlling for personal best time in a 100 km ultra-marathon as a potential confounder for the difference between 100 km race times revealed that the resulting mean (SE) race time difference of 5.5 (±28.6) min. remained no longer statistically significant when adjusted for the personal best time in a 100 km ultra-marathon. Personal best time proved to be an important variable regarding performance in ultra-endurance races [37]. Thus, adjusting for personal best time resulted in a non-significant difference in race time between the two groups.

The number of athletes might also have affected the result. A decrease of 0.6 kg in body mass seems to be relevant. In a recent study of male 100 km ultra-marathoners, skeletal muscle mass decreased by 0.7 kg [2]. Regarding statistical power, we would have needed to include 42 subjects per group to detect a clinical relevant difference between the groups of 80% power. With our actual sample size, we had only 60% power. However, it was not possible to increase the sample of athletes under field conditions since only these 28 ultra-marathoners from the total field of athletes volunteered to participate.

Since variables of skeletal muscle damage, such as creatine kinase and myoglobin, remain increased for up to seven days after a marathon [38], they should be measured not only immediately after the race but also in the recovery phase. Presumably the intake of amino acids during the race would lead to lower values of creatine kinase and myoglobin in the recovery phase.

In a multi-stage ultra-endurance run, skeletal muscle mass decreased continuously throughout the race [11,12]. Presumably, amino acid supplementation would have an effect on variables of skeletal muscle damage rather in a multi-stage race than in a single ultra-marathon. It has been shown that the oral administration of amino acids resulted in a faster recovery of muscle strength after eccentric exercise [39]. The ingestion of protein during rest periods might enhance recovery [40]. In runners, especially, the combined ingestion of carbohydrate and protein after each training session over 6 days reduced the post exercise increase in serum creatine kinase and muscle soreness [34].

## Conclusions

The ingestion of 52.5 g of amino acids immediately before and during a 100 km ultra-marathon had no beneficial effect on variables of skeletal muscle damage, muscle soreness, and race performance. A positive effect of amino acid supplementation in ultra-runners might be expected when amino acid or protein would be supplemented in the rest period during a multi-stage ultra-endurance run. Recovery might be enhanced and increase in variables of skeletal muscle damage might be reduced, effects that should be investigated in future studies.

## Competing interests

The authors declare that they have no competing interests.

## Authors' contributions

BK designed the study and wrote the manuscript. PK and CM carried out blood analysis and assisted the manuscript preparation. OS was responsible for statistical analysis and manuscript preparation. TR assisted the design of the study and manuscript preparation. RI and PB assisted in data analysis, data interpretation and manuscript preparation. All authors have read and approved the final manuscript.
